# Knowledge and Preventive Practices as Potential Factors of Empowerment Against Dengue in Córdoba, Colombia

**DOI:** 10.1155/ipid/6650290

**Published:** 2026-10-12

**Authors:** Jorge Eliecer Ortega Montes, Nydia Nina Valencia Jiménez, Elsy Cecilia Puello Alcocer

**Affiliations:** ^1^ Department of Social Sciences University of Córdoba Montería Córdoba Colombia uco.es; ^2^ Department of Nursing University of Córdoba Montería Córdoba Colombia uco.es

**Keywords:** dengue, empowerment, interdisciplinary studies, sociodemographic factors

## Abstract

**Introduction:**

Given the growing health impact of dengue fever in recent years, which primarily affects Latin American countries and Colombia in particular, interdisciplinary studies and scientific and technological advances to combat it, such as vaccines, mosquito genetic modification techniques, vector control using drones, and digital surveillance systems, among others, are becoming increasingly important. However, despite these achievements, little is known about the disease itself. The relationship between knowledge, sanitation conditions, and the preventive actions against dengue can be understood as part of the whole process of construction of community empowerment. Rather than acting solely as external factors, these dimensions constitute interrelated components that reflect the individuals and communities’ capacity to understand, confront, and actively participate in dengue prevention and control. Against this background, the main contribution of this study lies in addressing community empowerment in the face of dengue as a multidimensional construct comprised of dimensions of knowledge, sanitation, and preventive practices, allowing us to explore its internal structure and understand how these dimensions relate within a common analytical framework.

**Method:**

A quantitative approach with an observational, cross‐sectional, and analytical design was used. A total of 352 people infected with dengue during 2019 in the department of Córdoba, Colombia, were surveyed. Each response was assigned a score using the Likert scale question technique. Subsequently, a composite empowerment index was constructed. This index was conceptualized as a multidimensional construct consisting of domains related to knowledge, sanitation, and preventive practices against dengue. Consequently, the analysis did not aim to evaluate external predictive relationships between these components and empowerment, but rather to explore the relative contribution of the dimensions that internally comprise the construct and its association with different levels of empowerment.

**Results:**

The analysis revealed differences in the relative contribution of the components making up the empowerment construct. The higher index levels were associated with higher scores in the dimensions related to knowledge and preventive practices. In contrast, profiles characterized by favorable sanitation conditions, but lower levels of knowledge and preventive skills, tended to be located in lower categories of the empowerment index. This indicates that there is heterogeneity in the internal construct structure and reflects the relative importance of its constituent dimensions.

**Conclusion:**

It is concluded that empowerment has to be understood not only as the result of an individual effort or purpose but also as a complex and multidimensional process whose levels of development reflect the individual, relational, and collective capacity to act together and achieve significant changes.

## 1. Introduction

Dengue is one of the tropical diseases with the greatest impact on health, so much so that nearly half of the world’s population is at risk of infection[1]. However, during certain periods, it is enhanced in both endemic and nonendemic regions to the point of being declared a health emergency [2], as occurred in 2023, when the Pan American Health Organization (PAHO) and the World Health Organization (WHO) reported an increase in the number of cases (4.1 million) in the Region of the Americas, being the highest historical record since 1980. In 2024, 12.6 million cases were reported, with Colombia being one of the most affected countries, along with Argentina, Brazil, and Mexico, concentrating 90% of the cases and 88% of the deaths from this disease [3].

The epidemiological behavior of dengue in Colombia reveals a growing trend with high incidence rates during the epidemic cycles of 2010, 2013, 2016, 2019, and 2023. In 2019, for example, an expected epidemic phase began based on the cyclical behavior of the event, exceeding records compared to 2016 (313.5 cases per 100,000 inhabitants), with the department of Córdoba standing out as one of the regional entities with cases registered in more than 40 epidemiological weeks [4].

The WHO explains that much of the increase in dengue is due to the effects of climate change, expressed in excessive rainfall, high humidity, and rising temperatures [2]. Likewise, factors such as unplanned urbanization, human migration, inadequate basic services, solid waste collection and treatment, and water storage practices [1] constitute risk factors that must also be considered, along with the knowledge, attitudes, practices, and participation of communities, to address this disease, as suggested by the WHO itself during the First International Conference on Health Promotion, held in Ottawa, Canada, in 1986 [1, 4–6].

Ever since then, as is known, efforts to prevent and control dengue have been intensified, with the evolution of the concepts of health and empowerment under a human rights and development approach being highlighted, focusing on the actions in the following fundamental axes: epidemiology, integrated vector management, laboratory areas, patient care, communication and environmental monitoring, operational research, and skills development; emphasis on the active participation of individuals and families in monitoring and controlling risk factors as part of the empowerment process [7, 8].

However, periodic evaluations conducted by the Integrated Management Strategy for the Prevention and Control of Dengue in the Latin American Region (EGI‐Dengue) reveal multiple limitations in the implementation of some of these components, hindering the success and effectiveness of this strategy [7]. This situation requires strengthening health systems by developing institutional capacities to provide relevant education to individuals, families, and communities, tailored to their learning needs.

In the context of Colombia, especially in the department of Córdoba, the implementation of this strategy registers ambiguous results in controlling the spread of the disease, presumably due to the deficit in the mobilization of knowledge, insufficient participation, and scarce intersectoral and extra‐sectoral management that restricts local capacities to promote family and community empowerment [9, 10].

Thus, empowerment practices are affected by multiple factors that hinder informed decision‐making for proper control and prevention. In short, regarding health, empowerment is assumed to be more about self‐care and individual responsibility, where people are considered mere recipients of care who have needs and rights that must be defended by an external agent, and not as a cognitive, affective, and behavioral process in which people, organizations, and communities learn and contribute to solving their problems and transforming their living conditions [11].

Consequently, in this department, dengue has been little addressed from an empowerment perspective, as evidenced in much of the recent research with a predominance of some approaches focused on predictive methods based on climatic conditions or predictive and prescriptive models for the clinical management of the disease [12, 13], while others address susceptibility to insecticides or experimental research with animals [14, 15]. This circumstance justifies the intent of this article, which proposes to explore the relative contribution of knowledge on dengue, preventive practices, and housing sanitation conditions, as well as the association between these dimensions and the levels of empowerment of people or communities affected by the disease.

Based on the above, this study aims to analyze the relationship between some sociocultural and contextual aspects as factors of community empowerment, emphasizing the dimensions of sanitation, knowledge, and preventive practices, and including sociocultural variables such as sex, age, marital status, ethnicity, educational level, housing, family composition, and economic income.

This analysis is based on the understanding that empowerment, within this context, is a key factor in the processes of strengthening health and social protection, which involves providing communities with tools related to education, information, and resources so that they can actively participate in their health care and influence health policies to autonomously manage their social and economic well‐being [16], and, under the assumption that health education and knowledge about dengue are closely interrelated and/or conditioned by sanitation levels, which in turn affect the probability of empowerment.

## 2. Method

### 2.1. Study Design

A quantitative approach with an observational, cross‐sectional, and analytical design was used to evaluate community empowerment in the prevention and control of dengue. Empowerment was addressed as a multidimensional construct comprised of dimensions related to knowledge, preventive practices, and sanitation. Moreover, sociocultural characteristics such as sex, marital status, ethnicity, educational level, housing characteristics, family composition, and economic income were considered to describe and analyze the profiles observed in the studied population.

### 2.2. Research Context

The study was conducted in the department of Córdoba in the Colombian Caribbean region (Figure 1), which has an estimated population of 18,98,911 inhabitants distributed in 30 municipalities [17].

**FIGURE 1:**
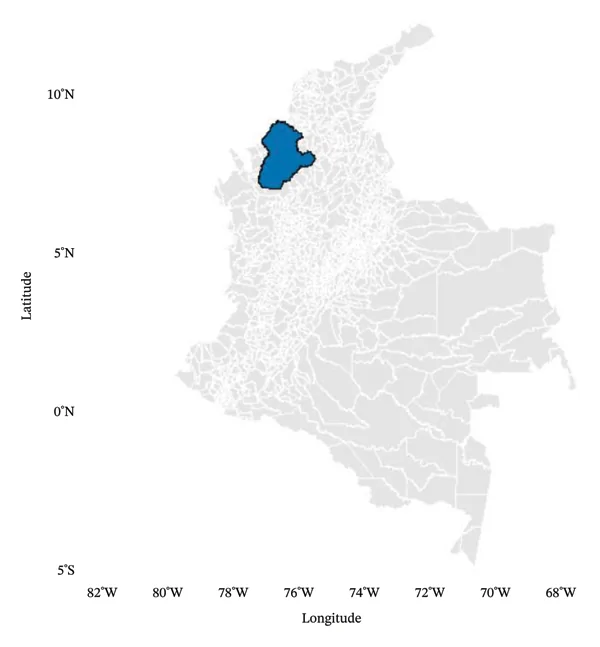
Department of Córdoba in Colombia.

### 2.3. Population and Sample

The target population consisted of 4169 people diagnosed with dengue during 2019 in the department of Córdoba [18], from which a sample of 352 people was determined using a multiple‐step sampling.

In the first stage, the municipalities of Montería, Pueblo Nuevo, San Andrés de Sotavento, and San Bernardo del Viento were selected (Figure 2); in the second stage, inside of each municipality, an urban and rural zoning to delimit and select communes, neighborhoods, districts, and rural sectors was conducted. In the third stage, the households were identified and randomly selected, using them as primary sampling units. Subsequently, within each household, existing families were identified, and one was selected. Finally, within the selected household or family, the adult with history of dengue was identified; if this person was not available at the moment of the visit, an adult over 18 years of age who was present during the survey application was selected.

**FIGURE 2:**
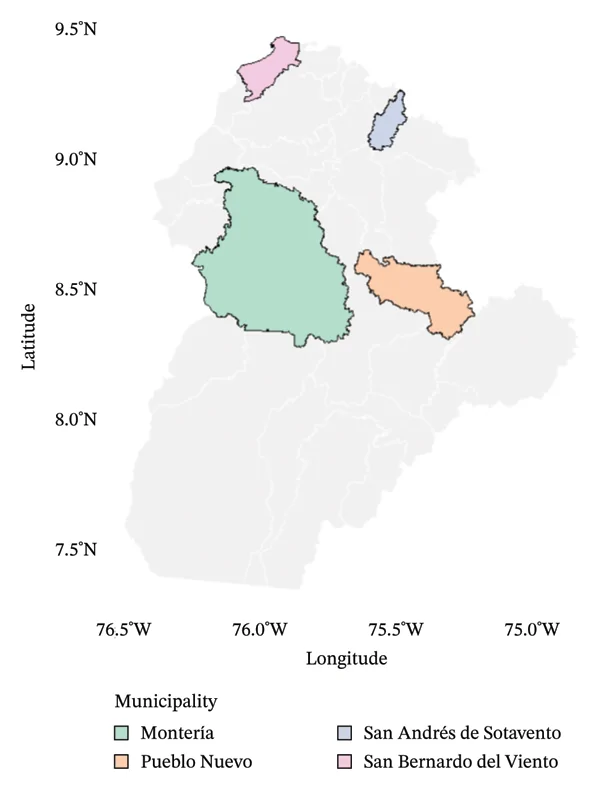
Selected municipalities in the study in the department of Córdoba.

The selection criteria that guided the decisions regarding the four municipalities were as follows: Montería, for having the highest number of cases reported by the National Public Health Surveillance System (SIVIGILA); Pueblo Nuevo, for having the highest incidence rate per 1000 inhabitants; San Andrés de Sotavento, for its indigenous majority; and San Bernardo del Viento for its predominantly Afro‐descendant population [18].

To estimate the sample size, the prevalence of dengue in the at‐risk Colombian population in 2023 was first considered, resulting in a minimum sample size of 254 individuals, with a 99% confidence level and a 1% margin of error. However, the total number of individuals surveyed reached 352, exceeding the initially estimated size under the established assumptions. Complementarily, it was observed that the sample size achieved was sufficient.

For sample selection, the inclusion criteria were defined as follows: eligible participants had to (a) be over 18 years of age; (b) have permanent residence in one of the four municipalities selected to participate in the study; (c) be registered in the SIVIGILA database as a person with dengue in Córdoba in 2019; and (d) not have changed residence in the last two years. The following exclusion criteria were also applied: (a) being under 18 years of age; (b) having temporary residence in the locations selected for the study; and (c) suffering from physical, psychiatric, or emotional conditions that limit their ability to answer the survey questions.

### 2.4. Data Collection

Data were collected using a questionnaire administered in person, house by house. The questionnaire consisted of five components with 37 questions: (i) sociocultural characteristics, (ii) knowledge about dengue and preventive practices, (iii) sanitation, (iv) participation, and (v) governance. For the purposes of this article, the participation and governance components were excluded from the analysis.

The instrument underwent content validity testing through expert review by specialists in public health, epidemiology, community nursing, and social psychology. A pilot test was also conducted with 30 participants with characteristics similar to the sample to identify gaps or strengths in the comprehension of the questions.

Additionally, the instrument underwent validity and reliability testing. A Cronbach’s alpha coefficient of 0.879 was obtained, indicating adequate internal consistency. Exploratory factor analysis yielded a Kaiser–Meyer–Olkin (KMO) measure of sampling adequacy of 0.597, and Bartlett’s test of sphericity was significant (*p* < 0.005), demonstrating acceptable conditions for applying the factor analysis. However, the KMO value obtained was close to the lower recommended limit, an aspect that is interpreted as a limitation for the structural validity of the instrument.

Participants were initially contacted by telephone to coordinate their availability. During the face‐to‐face survey process, each person’s willingness to participate was confirmed, the informed consent was read, and after their acceptance, the information was collected by asking the questions in the questionnaire.

### 2.5. Study Variables

The dependent variable of the study was the level of empowerment, conceptualized as a multidimensional construct and operationalized by means of a composite index obtained from the sum of the scores obtained in the following dimensions: knowledge of the disease (concepts, causes, symptoms, and treatment, maximum 8 points), sanitation practices in the household (infrastructure and sanitary services), and preventive practices against the disease (11 points).

The independent variables corresponded to the participants’ sociocultural characteristics (sex, marital status, age, socioeconomic level, educational level, family structure, social stratum, ethnicity, religion, and income). The sanitation dimension of the household assesses the basic conditions of access, storage and handling of water, waste disposal and hygiene and individual prevention practices, related to the availability and frequency of drinking water supply, the existence of sewerage systems, the periodicity of solid waste collection, and the actions and the use of elements for the mosquito proliferation control at home and in its surroundings.

The knowledge dimension explores participants’ understanding of dengue fever, its transmission methods, symptoms, and associated causes.

### 2.6. Data Management and Analysis

Empowerment was measured using a Likert‐type questionnaire that included different conceptual dimensions related to the respondents’ knowledge, sanitation, and preventive practices. Each item was assigned an ordinal score according to the level of complexity of the response: none (0), basic (1), medium (2), and high (3). An overall empowerment index was constructed from the total sum of the item scores.

The cutoff points for classifying empowerment into low, medium, and high levels were established based on the proportion of the maximum possible score (≤ 25%, 25%–75%, and ≥ 75%), following criteria reported in previous studies on dengue and health empowerment [19, 20]. This procedure is based on the principles of classical test theory, where the sum of items represents the magnitude of the latent trait being assessed [21].

Regarding socioeconomic status, the official classification used in Colombia was employed, which groups households into six categories (1–6) according to the physical conditions of the dwelling and the environment, with stratum 1 being the lowest socioeconomic level and stratum 6 the highest.

For inferential analysis, an ordinal regression model (proportional logit) was used, considering the level of empowerment categorized into three ordered levels as the dependent variable. Initially, the association between sociocultural variables and empowerment was explored using Pearson’s chi‐squared test, with a statistical significance level of *α* = 0.05. The independent variables were continuous numerical variables, represented by the sum of points obtained in each of the dimensions or scales (sanitation, knowledge, and preventive practices).

This approach allowed modeling the cumulative probability of belonging to a higher level of empowerment based on the scores obtained in the dimensions analyzed, maintaining consistency with the ordinal nature of the dependent variable and the proportional odds model assumptions.

The results showed no statistically significant associations or consistent patterns between sociocultural variables and the level of empowerment across the different municipalities analyzed. Consequently, these variables were retained solely for descriptive purposes to characterize the population context and were not included in the final multivariate model.

In the inferential stage, an ordinal regression model was fitted to analyze the relative contribution of the dimensions that make up the empowerment index regarding the different levels of this construct. The model was specified considering the ordinal level of empowerment (low, medium, and high) as the dependent variable and the scores obtained in the dimensions that showed consistent and significant relationships with this variable. Specifically, these dimensions included sanitation, knowledge, and preventive practices, understood not as external predictors, but as internal components of the construct.

An important methodological consideration regarding the level of empowerment in this study is that it was operationalized via a composite index comprising the dimensions of knowledge, sanitation, and preventive practices—all of which were subsequently included in the adjusted ordinal model. Consequently, the resulting coefficients should be interpreted as the relative contribution of each dimension to the construct’s internal structure and the scoring system used to create it, rather than as independent associations between conceptually distinct variables. Therefore, the results should not be viewed as evidence of external predictive relationships, but rather as an analysis aimed at describing the internal behavior of the empowerment index used in this study.

Future research could assess empowerment using external indicators or conceptually distinct independent variables, thereby allowing for an analysis of factors associated with empowerment that is free from the dependency inherent in the operationalization employed here.

The information was processed by creating a database using the software Excel (Microsoft 365), R Studio Version 4.4.6, and SPSS (Statistical Package for the Social Sciences, Version 25).

### 2.7. Ethical Approval of Research

The study was conducted in accordance with the Declaration of Helsinki and was approved by the Institutional Research Committee of the University of Córdoba under code FE‐11‐19, issued in 2021. The criteria of Resolution 8430 of 1993, which establishes the scientific, technical, and administrative standards for health research in Colombia, were adopted, assuming within the ethical criteria the written informed consent of all participants and guaranteeing their anonymity.

## 3. Results

The people surveyed are, on average, 41 years old (with a standard deviation of 14.2 years); 75.6% of respondents are women, while men represent 24.4%. A total of 81.3% come from urban areas and 18.8% live in rural areas; Montería leads with 41.2% of respondents, followed by Pueblo Nuevo (25.0%), San Andrés de Sotavento (17.9%), and San Bernardo del Viento (15.9%).

The majority of respondents are linked to the subsidized regime (83.8%), an indicator that reinforces the predominant socioeconomic condition of stratum 1, according to the System for Identifying Potential Beneficiaries of Social Programs (SISBEN) in Colombia, since it was found that a large part of the sample belongs to this stratum (87.2%); these data are consistent with the fact that 67.6% of households report incomes less than one current legal minimum wage (SMMLV), and an additional 17.3% have incomes equivalent to one SMMLV; only 1.1% have incomes that range between two and three SMMLV.

The number of people in households is also a relevant indicator: 63.9% of households have between three and five people, while 26.5% have six or more members. This suggests that many of these families live in overcrowded conditions; almost half of those surveyed (48.3%) report being in unpaid work.

The educational level of the participants shows that 32.4% have completed high school, making this the most common category. However, 5.1% are identified as illiterate. Access to higher levels of education, such as technician (11.6%), technologist (2%), professional (6.5%), or specialist (0.6%), is limited.

### 3.1. Association Between Sanitation, Sociocultural Characteristics, and Housing

The “sanitation” dimension includes preventive practices in the home, infrastructure, and sanitary services in the home (11 points), the figure of which was obtained from the sum of the items corresponding to each of the answers obtained in the survey questionnaire.

When applying the chi‐square test to study the associations, a statistically significant association was identified between origin and sanitation level (*p* = 0.0020), observing that people residing in urban areas have a higher proportion in medium and high sanitation levels, while those living in rural areas are mainly concentrated in the lowest level. This is reaffirmed by the level of association between the municipality of residence and the sanitation level (*p* < 0.0001), such as the inhabitants of Montería, standing out with a high concentration in the medium and high levels, an aspect that suggests that access to better sanitary conditions is linked to the urban environment, where it is more feasible to have adequate infrastructure and services.

For its part, ethnicity shows a highly significant relationship (*p* < 0.0001) with the level of sanitation, predominantly with the low level, as does the condition of belonging to special populations, such as people in a situation of displacement or victims of conflict (*p* = 0.0070). In contrast, the variables of sex, social security, educational level, and religion did not show a statistically significant relationship with the level of sanitation.

Significant associations were also observed between sanitation levels and housing conditions (*p* = 0.0340), as well as the number of people in the household (*p* = 0.0320). Households with one or two members were more prevalent in the middle and high levels, while those with six to eight people were mainly concentrated in the low level, indicating that a larger number of occupants is associated with more precarious sanitary conditions.

For their part, the variables socioeconomic status, occupation, and income did not show a statistically significant relationship with the level of sanitation (*p* > 0.05). This indicates that, in this analysis, there was insufficient evidence to conclude that socioeconomic status, type of employment, or income directly influences the spatial distribution of sanitation infrastructure in the territory and the internal sanitation conditions of housing.

### 3.2. Association Between the Level of Knowledge and the Main Sociocultural Characteristics

The level of knowledge was calculated from the sum of the items corresponding to this dimension within the questionnaire, on aspects related to individual prevention and the identification or management of dengue (concepts, causes, symptoms, and treatment = maximum 8 points).

As a result of applying the bivariate chi‐square test, a significant association between sex and the level of knowledge (*p* = 0.0400) was identified, with a higher proportion of women at the high level of knowledge compared to men. Origin also showed a significant relationship (*p* = 0.0360), demonstrating that people residing in urban areas have higher levels of knowledge than those from rural areas. Similarly, ethnicity was significantly associated with low level of knowledge (*p* ~ 0.001).

In contrast, the variables marital status, health insurance affiliation, educational level, religion, and belonging to a special population did not show statistically significant associations (*p* > 0.05). Socioeconomic status did show a significant relationship with the level of knowledge (*p* = 0.0050), with a higher proportion of higher levels observed compared to other strata. The variables number of families in the household, number of people in the household, occupation, and income did not show significant associations.

### 3.3. Association Between Empowerment and the Main Sociocultural Characteristics and Housing

Statistically significant differences were observed in the level of empowerment according to the municipality (*p* ~ 0.001). Montería showed a higher concentration of individuals with high levels of empowerment, compared to San Andrés de Sotavento (indigenous population), which showed a significant association with empowerment (*p* = 0.0010), but with a low level.

Among the other characteristics analyzed, only socioeconomic stratum one (1) showed a statistically significant association (*p* = 0.0030), concentrating most of the low and medium levels of empowerment. Variables such as the number of people in the household, occupation, and income did not show a significant relationship with the level of empowerment (*p* > 0.05).

### 3.4. Ordinal Analysis of the Contribution of the Dimensions to Empowerment

An ordinal regression model was fitted in which the dependent variable was the level of empowerment, categorized into three ordered levels (1: low, 2: medium, and 3: high). The variables included in the model corresponded to the scores obtained in the dimensions of sanitation, knowledge, and preventive practices, considered as internal components of the empowerment index, with the objective of exploring the relative contribution of these dimensions to the internal structure of the construct and examining its relation with the different empowerment levels observed.

The model, specified using the cumulative logit function, showed that all three dimensions had statistically significant associations with the level of empowerment (*p* < 0.05)*.* Sanitation showed a negative association, while knowledge and preventive practices showed positive associations with higher levels of empowerment (Table 1).

**TABLE 1:** Model coefficients.

Variable	Coefficient	Standard error	T‐value	Interpretation
Sanitation	−0.1051	0.0532	−1.9750	Higher sanitation scores were associated with relatively lower categories of empowerment within the operationalization used in the study.
Knowledge	0.9201	0.0884	10.4100	Higher knowledge scores were associated with higher categories of empowerment.
Preventive practices	0.7601	0.0920	8.2610	Higher scores on preventive practices were associated with higher categories of empowerment.

*Note:* Source: Own work.

The estimated cutoff points (Table 2) allow us to identify the thresholds between the ordinal categories, while the fit indicators (AIC = 528.78; residual deviance = 518.78) confirmed an adequate fit of the model. Moreover, Brant’s test confirmed compliance with the proportional odds assumption both globally (*p* = 0.85) and for each of the dimensions included, which supports the consistency of the interpretation of the model.

(1) logPY≤jPY>j=β∧0j+β∧1·Sanitation+β∧2·Knowledge+β∧3·Preventive practices.

**TABLE 2:** Estimated thresholds (intercepts) between the empowerment categories.

Threshold	Coefficient	Standard error	t‐value
Low | medium	9,1013	0.8254	11,0265
Medium | high	11.5216	0.9329	12,3508

*Note:* Residual deviance = 518.78; AIC = 528.78.

Source: Authors’ own elaboration.

The coefficients estimated for the dimensions of sanitation (*t* = −1.98), knowledge (*t* = 10.41), and preventive practices (*t* = 8.26) indicate that the predictors are statistically significant at a 5% level (*p* value < 0.05). In the adjusted model, sanitation presented a negative association, while knowledge and preventive practices showed positive associations with the empowerment categories, according to the operationalization used in the study.

The low/medium point of 9.1013 indicates the limit where the probability of being at a “low” level is equal to that of being at a “medium” or “high” level. The medium/high point of 11.5216 indicates the limit where the probability of being at a “medium” level is equal to that of being at a “high” level.

#### 3.4.1. Significance Test (ANOVA)

The analysis of variance showed that sanitation, knowledge, and preventive practices were significantly associated with the empowerment category. The sanitation variable had a *p* value of 0.0468, while knowledge and preventive practices had *p* values below 0.0001, indicating statistically significant associations with empowerment.

When applying the multicollinearity test (variance inflation factor [VIF]), in the variables of sanitation (1.2105), knowledge (1.3382), and preventive practices (1.1582), VIF values lower than 5 were found, indicating that there are no multicollinearity problems between these variables; therefore, the predictors are not highly correlated with each other.

The odds ratio analysis showed that sanitation presented an OR of 0.9002, indicating that an increase in one unit of this variable was associated with lower probability of being in a higher empowerment category, with a relative probability reduction of about 9.98%. Knowledge, on the other hand, showed an odds ratio of 2.5096, indicating that an increase of one unit of knowledge is associated with a higher empowerment category by 250% within the operationalization used. Similarly, preventive practices showed an odds ratio of 2.1385, indicating that a one‐unit increase in preventive practices is associated with higher empowerment categories, specifically to an increase of 113.85%.

In light of this, the model was found to be statistically significant, as knowledge, sanitation, and preventive practices showed statistically significant associations with the empowerment categories. Specifically, greater level of knowledge is associated with higher estimated probability or higher odds of being in a higher empowerment category (OR = 2.51; *p* value < 0.05), while a higher level of sanitation is associated with lower odds of pertaining to a higher category (OR = 0.90; *p* value < 0.05). Likewise, having more preventive practices is also related to higher odds of pertaining to higher empowerment categories (OR = 2.14; *p* value < 0.05). No multicollinearity issues were found when the VIF was below 5.

The omnibus test, with an associated *p* value of 0.85, indicates that there is no evidence to reject the null hypothesis; that is, the assumption of proportional odds in the variables is met. Furthermore, for the individual variables sanitation (*p* value = 0.98), knowledge (*p* value = 0.40), and preventive practices (0.72), there is no evidence that the model violates the proportionality of odds assumption; that is, the model is adequate and valid for interpretation.

For this case, the adjusted model is

(2) logPY≤1PY>1=9.10130.100.920.76−·Sanitation+·Knowledge+·Preventive practices,

(3) logPY≤2PY>2=11.52160.100.920.76−·Sanitation+·Knowledge+·Preventive practices,

where

• *P*(*Y* ≤ 1) is the probability that empowerment will be “low.”
• *P*(*Y* ≤ 2) is the probability that empowerment will be “medium” or lower.
• *P*(*Y* > 1) is the probability that empowerment will be “medium” or higher.
• *P*(*Y* > 2) is the probability that empowerment will be “high.”
• ${\overset{\wedge }{\beta }}_{1}=-0.10$—a higher level of sanitation decreases the likelihood of being at a high level of empowerment.
• ${\overset{\wedge }{\beta }}_{2}=0.92$—a higher level of knowledge increases the likelihood of being at a high level of empowerment.
• ${\overset{\wedge }{\beta }}_{3}=0.76$—a higher level of preventive practices increases the likelihood of being at a high level of empowerment.
• 91, 013 and 1, 15, 216 are the cutoff thresholds (intercepts) that separate the ordinal categories.

#### 3.4.2. Illustrative Scenarios Derived From the Ordinal Model

For purely illustrative purposes, Table 3 presents several scenarios derived from the ordinal model, fitted using various combinations of scores across the dimensions of knowledge, sanitation, and preventive practices. Since these dimensions constitute the components used to operationalize the empowerment index, the resulting probabilities reflect the internal behavior of the model’s scoring structure and should not be interpreted as estimates of independent effects or predictive relationships between distinct variables.

**TABLE 3:** Applications of the ordinal model.

Case	Scores			Probability		
	Sanitation	Knowledge	Skills	Low (%)	Half (%)	High (%)
1	7	1	2	99.94	0.05	0.01
2	2	8	5	13.58	50.30	36.12
3	2	9	6	2.85	21.94	75.21
4	1	10	9	0.11	1.09	98.80

*Note:* Source: Own elaboration.

In Case 1, a sanitation score of 7 (medium), together with a knowledge score of 1 and a preventive practices score of 2 (both low), was associated with a predicted probability of 99.94% for the low empowerment category in the adjusted model. The corresponding probabilities were 0.05% for medium empowerment and 0.01% for high empowerment. In Case 2, a sanitation score of 2 (low), combined with a knowledge score of 8 and a preventive practices score of 5, was associated with a predicted probability of 50.30% for the medium empowerment category.

In the third case, the combination of a sanitation score of 2 (low), knowledge of 9 (high), and preventive practices of 6 (medium) produces an estimated probability of high empowerment of 75.21%. In this context, if we consider a sanitation score of 1 (classified as low), a knowledge score of 10, and a preventive practices score of 9 (classified as high), an estimated probability of high empowerment of 98.80% is presented. These results correspond to the scenarios evaluated within the adjusted model and reflect the behavior of the scoring structure used.

## 4. Discussion

This research provides evidence on dengue prevention from an empowerment perspective, based on an integrated analysis of the dimensions of knowledge, sanitation, and preventive practices. The findings show that higher levels of knowledge and preventive practices are associated with higher levels of empowerment, while sanitation shows an inverse relationship, which may reflect characteristics of the scoring structure or the distribution of scores for the components of empowerment.

In addition, people living in conditions with limited access to basic services, such as water supply, sewage, and waste collection, showed higher levels of knowledge and preventive practices in the present study. This finding may reflect a greater perception of dengue‐related risk and contrasts with the assumption that poorer living conditions necessarily correspond to lower preventive capacity. Previous studies have shown that dengue prevention and control are multifactorial processes involving individual, family, and community knowledge, attitudes, and preventive practices, as well as community participation, empowerment, and organizational capacity [22–25].

Hence, it is important to propose this type of study, given the need to generate new ideas and information from the territories that contribute to the formulation and management of public policies and the reorientation of health programs [9].

While some sociocultural characteristics, such as sex, socioeconomic status, and place of origin, showed statistically significant associations with levels of knowledge, sanitation, and empowerment in bivariate analyses, these variables were used for descriptive and exploratory purposes, without the development of an explanatory model that positions them as determining factors. In this context, it should be highlighted that women have higher levels of knowledge about dengue compared to men, such as previous studies describing greater female participation in health‐related activities within the family and community [24, 25]. However, this finding does not imply that gender is an independent predictor of empowerment, but rather reflects descriptive differences in the distribution of knowledge within the analyzed population.

The above suggests that, in this context, having women or people with technical or secondary education in health programs is beneficial because they may be better prepared to actively engage in community‐based interventions to prevent disease.

Similarly, although associations were identified between socioeconomic status and levels of knowledge and empowerment, these should not be interpreted as direct effects of status, but rather as expressions of structural inequalities that coexist with other contextual factors not modeled in the study. In contrast, the level of education did not show significant associations with knowledge or sanitation, which differs from what has been reported in studies conducted in other contexts, such as Saudi Arabia [26], and reinforces the idea that knowledge about dengue does not depend exclusively on formal education.

Moreover, it is observed that knowledge and attitudes are a necessary component for dengue prevention, which coincides with the results reported in Sri Lanka, which demonstrate how knowledge and attitudes are an essential step in behavior modification for disease risk reduction [23], but they do not always translate into effective practices [27]. Thus, the relevance of empowerment as a multidimensional process that integrates not only access to information but also the capacity to make decisions, self‐confidence, and the real possibility of acting in specific contexts [8] should be emphasized.

From this perspective, the need arises for rethinking health education programs in different sectors [25] as a strategy to promote empowerment, as well as the creation of simple and accessible messages for all audiences, including the rural population [28], which is at a disadvantage in becoming empowered, as demonstrated in the previous findings.

Under this premise, it is valid to consider education that promotes health not only as a means for promoting motivation for disease prevention and control but also as a tool that generates the behavioral and attitudinal change required in empowerment processes, even more so because as it achieves the goal of increasing knowledge about dengue, the chances of people making better decisions about their health increase [9], without this implying an increase in the level of empowerment.

These research findings demonstrated that knowledge alone, while an influential factor, is not sufficient to increase the level of empowerment. This situation suggests that if health education were implemented as a communicative action based on social interaction for mutual understanding and the search for conceptual, strategic, and operational consensus regarding prevention and control programs, more satisfactory results could be obtained [6], as this would strengthen individual and collective awareness for community participation [8].

Another point to consider is that, while socioeconomic status, occupation, and income did not show a statistically significant relationship with sanitation levels in the Pearson chi‐square test (*p* > 0.05), this condition does not directly influence the spatial distribution of sanitation infrastructure or the quality of public utilities such as water supply, sewage, and sanitation. The same applies to the variables number of families in the household, number of people in the dwelling, occupation, and income, which did not show a significant relationship with empowerment levels (*p* > 0.05), but rather are associated with sanitation levels, as a greater number of households and people in the dwelling reflect worse conditions of infrastructure, quality, and access to sanitation services (*p* = 0.0340, *p* = 0.0320).

The above supports the hypothesis that empowerment is a complex process that integrates different dimensions and/or factors. It is built through a combination of the following dimensions: subjective, which integrates personal beliefs and self‐confidence; relational and collective; access to information; and decision‐making skills [8].

Considering the potentialities, barriers, and opportunities, the context offers for empowerment against dengue, the findings regarding sanitation reflect the gap and inequality that currently characterize the Colombian population in this regard. According to the 2020 national census of the National Administrative Department of Statistics (DANE), 88% have access to drinking water; however, a large part of this access corresponds to the municipal capitals (97.4%), leaving rural communities at a disadvantage. Likewise, according to the same agency, 88.1% of this population has sanitation services, with 91.9% of this total corresponding to urban areas and 76% to rural areas [17].

This situation reinforces the differences in perception observed in this regard: in areas with greater sanitation deficiencies, the population perceives itself to be at higher risk of dengue and may, as a result, develop greater knowledge and more frequently adopt preventive practices—whether promoted by public health programs or driven by accumulated experience with the disease. It may also reflect the internal structure of the scoring system used and the relative distribution of scores across the adopted dimensions, rather than an independent inverse relationship between sanitation and empowerment.

Therefore, rather than being directly interpreted as evidence that better sanitation conditions reduce community empowerment, this finding represents a hypothesis to be confirmed in future research on the subject.

Taken together, all of these are risk factors and conditions related to the prevalence of dengue in Colombia, which, according to Ortiz et al. [29], are exacerbated by land tenure issues, migration processes, and forced displacement. Therefore, it is necessary to reflect on the relevance of promoting a conceptual, epistemological, and methodological shift in the approach to dengue, which implies the adoption of holistic perspectives that incorporate all forms of knowledge, participation, and governance of the different groups and social actors within a territory, with a view to improving the living conditions of the population.

A vision with these characteristics could contribute to a more equitable distribution of public resources allocated to health infrastructure and the quality of water, sewerage, and sanitation services with a territorial approach, taking advantage of the human, cultural, and educational potential of the population, through the dialog of knowledge between ancestral, popular knowledge, and scientific and technological knowledge [10].

At the territorial level, one could consider planning and implementing control and prevention programs with differential and territorial approaches that promote understanding of health and disease processes by adults, male participation, improvement of family income, and greater public investment in endemic regions such as the department of Córdoba, which can improve sanitation services and reduce dengue risk factors.

Among the possible limitations of the study are the absence of multivariate models that allow for the joint evaluation of the effect of these sociocultural variables on empowerment, as well as the exclusion of other relevant social factors, such as community organizations and health institutions, and the role of women in healthcare both at home and in the social and community context, which implies delving deeper into those factors that impede and/or favor the development of their organizational and human potential.

Consequently, the findings of this study are relevant because, in addition to providing information for policymakers, they identify new challenges for academia, research, the community, and society in general, aimed at deepening the understanding of the social and community mechanisms that influence dengue prevention and the strengthening of collective empowerment.

## 5. Conclusions

Empowerment was examined in relation to housing sanitation conditions, people’s knowledge levels, and certain sociocultural characteristics, from an approach based on three general dimensions. The findings revealed differential associations between the dimensions that make up the empowerment construct. Knowledge and preventive practices showed positive relationships with higher levels of empowerment, while sanitation showed an inverse association within the operationalization used.

Conversely, differences were observed in the distribution of the analyzed dimensions according to contextual and sociocultural characteristics, with a higher concentration of certain profiles being identified in urban environments, such as Montería, the departmental capital, with socioeconomic stratum being one and women being the social actors that stand out for having the highest levels of knowledge and empowerment.

This situation partially confirms the proposed hypothesis, showing that empowerment to confront dengue is not the result of an individual desire or purpose, that is, it does not depend on a single dimension, but rather on a complex process integrating multiple factors and characteristics, and that should be understood as a multidimensional construct resulting from the interaction between individual, contextual, and social components.

Therefore, a paradigm shift is suggested for its understanding, in which the dengue epidemic is understood not only as a medical disease but also as a public health problem influenced by the environment, social factors such as poverty, and territorial factors such as urbanization, adopting a more holistic perspective that incorporates multiple forms of knowledge for its care and treatment.

## Funding

This study was funded by the University of Córdoba, under the code FE‐11‐19.

## Conflicts of Interest

The authors declare no conflicts of interest.

## Data Availability

The data that support the findings of this study are available from the corresponding author upon reasonable request. The data are not publicly available due to privacy and ethical restrictions.
